# Contributions of Subsurface Cortical Modulations to Discrimination of Executed and Imagined Grasp Forces through Stereoelectroencephalography

**DOI:** 10.1371/journal.pone.0150359

**Published:** 2016-03-10

**Authors:** Brian A. Murphy, Jonathan P. Miller, Kabilar Gunalan, A. Bolu Ajiboye

**Affiliations:** 1 Department of Biomedical Engineering, Case Western Reserve University, 10900 Euclid Avenue, Cleveland, OH, 44106, United States of America; 2 Department of Neurosurgery, Neurological Institute, University Hospitals Case Medical Center, 11100 Euclid Avenue, Cleveland, OH, 44106, United States of America; 3 Louis Stokes Cleveland VA Medical Center, 10701 East Boulevard, Cleveland, OH, 44106, United States of America; Wadsworth Center, UNITED STATES

## Abstract

Stereoelectroencephalographic (SEEG) depth electrodes have the potential to record neural activity from deep brain structures not easily reached with other intracranial recording technologies. SEEG electrodes were placed through deep cortical structures including central sulcus and insular cortex. In order to observe changes in frequency band modulation, participants performed force matching trials at three distinct force levels using two different grasp configurations: a power grasp and a lateral pinch. Signals from these deeper structures were found to contain information useful for distinguishing force from rest trials as well as different force levels in some participants. High frequency components along with alpha and beta bands recorded from electrodes located near the primary motor cortex wall of central sulcus and electrodes passing through sensory cortex were found to be the most useful for classification of force versus rest although one participant did have significant modulation in the insular cortex. This study electrophysiologically corroborates with previous imaging studies that show force-related modulation occurs inside of central sulcus and insular cortex. The results of this work suggest that depth electrodes could be useful tools for investigating the functions of deeper brain structures as well as showing that central sulcus and insular cortex may contain neural signals that could be used for control of a grasp force BMI.

## Introduction

Intracranial electrodes (stereo-electroencephalography, or SEEG), placed for clinical localization of seizures in patients with pharmacologically resistant epilepsy, offer a platform for understanding fundamental human neurophysiology, as well as investigating novel cortical areas for implanting electrodes for use in brain-machine interfaces (BMIs). In the present study, we use intracranial SEEG to first investigate the efficacy of using field potentials recorded from insular cortex and the intrasulcal wall of motor cortex to discriminate forces of hand grasping for controlling a BMI, and secondly to compare the modulations of the high (gamma) and low (alpha, beta) band activities from motor and insular cortices during grasp force production.

In contrast to subdural electrocorticography (ECoG) electrodes which record from the cortical surface, and intracortical microelectrodes which typically record from 1–1.5 mm deep into cortical tissue, SEEG depth electrodes can record neural activity from deeper cortical structures (such as insular cortex) that are not typically accessible by ECoG or intracortical arrays, and yet may exhibit movement-related modulation. Functional magnetic resonance imaging (fMRI) investigations have elucidated spatial patterns of activation during hand and finger movements that include activity in the central sulcus wall of motor cortex (MC) and the insular cortex (IC). Specifically, increased blood-oxygen-level-dependent (BOLD) signals have been observed inside the walls of central sulcus during hand and finger movements [1,2] and isometric force tasks [3]. Other imaging studies have shown movement-related activity in regions of the insular cortex during hand and finger movements [4,5]. Production of grasp force has also been associated with significant BOLD activation in deeper structures including the insula, precuneus, operculum, putamen, and globus pallidus [6–9]. Because of the strong relationship between BOLD activity and electrophysiological recordings (particularly gamma band activity) [10–12], recording of gamma, and possibly lower frequency band activities from intrasulcal MC and IC may provide relevant information for decoding hand grasp function in a BMI. Further support for the idea that there is movement and force related information inside of central sulcus comes from White et al. [13] who showed cytoarchitecturally that Brodmann Area 4 (primary motor cortex) is predominantly found inside of central sulcus rather than on the precentral gyrus and also from Yanagisawa et al. [14] who found modulation in intrasulcal electrocorticograms useful for classification of thumb, grasping or elbow movements. Finally, the insular cortex is of particular interest as a novel area of electrode implantation for upper-extremity BMI controlled neuroprostheses, as it has been implicated in contributing to the completion of motor tasks of the upper extremity and for motor learning [5,15], motor recovery [16], and hand-and-eye motor movements [4].

At a level deeper than discrimination of hand states, we sought to understand the temporal and spatial relationships between low (alpha, beta) and high (gamma) band activities in insular and motor cortices during hand grasp force production. Event-related synchronization (ERS) of the gamma band (30+ Hz) and event-related desynchronization (ERD) of the beta band (12-30Hz) have long been shown to be hallmarks of cortical sensorimotor activity during motor execution and even imagery. Yet it is unclear whether these same characteristic patterns of cortical modulation are evident in insular cortex, despite the reported contribution of insular cortex during movement generation. Beta band desynchronization occurs at the beginning of, and sometimes prior to, movement activity and is believed to be related to asynchronous activity of neurons within the cortical network as the neurons increase their firing rate. Beta desynchronization is also accompanied by synchronization in higher frequencies as more of the neurons start to fire at different rates, resulting in increased power in the high gamma frequencies [17]. Once the cortical system returns to a resting state, the beta band power increases in an event-related synchronization (ERS) sometimes even above resting levels in what is known as post-movement beta rebound as the neurons slow their firing rate and return to their baseline synchronized firing levels [18].

Several lower frequencies have been shown to have task related modulation as well. Modulation in the alpha frequency band (6–12 Hz) has been reported in EEG to respond to executed and imagined movement tasks [19] and isometric finger pinch forces [20]. The alpha frequency band has been shown to have both ERS and ERD responses to stimuli depending on the task and recorded brain region. Klimesch (2012)[21] suggested that the ERS state for the alpha band reflects a general inhibition of related neural circuits and ERD demonstrates a release from this inhibition. Delta band activity (0–2 Hz) can exhibit movement-related modulation during both executed and imagined wrist movements [22] and prediction of movement cues [23]. Finally, the local motor potential (LMP) [24] or low-frequency component (LFC) [25], which consists of the low-pass filtered (below 5 or 6 Hz) time-domain signal, has been shown to modulate with respect to hand grasp force production [26,27].

The present study reports comparative electrophysiological analysis of the relationships of neural activity from insular cortex and the intrasulcal wall of MC to production of hand grasp force. The findings show that insular cortex (at least in one participant) exhibits movement related cortical modulation, with focus in the alpha (6–12 Hz) frequency bands, and that this modulation alone is sufficient for discrimination of force execution vs non-force. Higher gamma frequency modulation (> 30 Hz) was not present in insular electrodes, in contrast to the clear gamma modulation observed in the intrasulcal wall of motor cortex. The present findings suggest that the motor contributions of insular cortex is fundamentally different than that of motor cortex, predominantly focusing modulation in the lower frequency band, rather than the higher frequency bands, and the observed modulations allows for motor discrimination during a hand grasp force task.

## Methods

### Participants and Electrode Localization

Four human participants (all male, ages 29–49 years) were enrolled into the study after providing written consent. The study was reviewed and approved by the Institutional Review Boards of Louis Stokes Cleveland VA Medical Center (IRB# 11068-H14) and University Hospitals Case Medical Center (IRB# 07-12-33). All study participants were patients at University Hospitals Case Medical Center (Cleveland, OH) admitted for epilepsy monitoring prior to resection surgery. Participants were implanted with SEEG electrodes from either Integra LifeSciences Corporation (Plainsboro, NJ) or PMT Corporation (Chanhassen, MN). Each Integra electrode consisted of 12 platinum-iridium macro-contacts measuring 1.1 mm in diameter and 2.3 mm in length, evenly spaced at 5 mm intervals. One participant had two PMT combination electrodes implanted consisting of 4 SEEG macro contacts with 4x6 circumferentially spaced microwires between the macro contacts (1.4mm macro contacts with 7mm center spacing). All participants underwent implantation of electrodes into the target structures exclusively for clinical purposes (i.e., delineation of the epileptogenic zone). Only electrodes passing directly through sensorimotor areas were used for this study. Study procedures were performed during non-epileptic episodes.

Each electrode contact was localized in 3-dimensional anatomical space in a custom Python-based visualization environment using the electrode contact artifacts in post-operative volumetric CT scans co-registered with the pre-operative MRI scans. Since the SEEG electrodes are fabricated from polyurethane tubing and the electrode contacts span 60 mm, the shaft of the electrodes can follow a curved course between superficial and deep targets. Thus, each contact was individually represented as a sphere and labeled by color according to the nearest neural structure. Using the T1-weighted image, FreeSurfer (http://surfer.nmr.mgh.harvard.edu/) [28] parcellated cortical regions of interest including the motor, premotor, primary sensory, and insular cortices [29] and to create 3D surface views of each participant’s brain. The supplementary motor area (SMA) was manually extracted from Freesurfer’s definition of the pre-frontal cortical region using FSLView (http://fsl.fmrib.ox.ac.uk/fsl/fslwiki/) [30]. We defined the SMA as the region on the medial surface of both hemispheres, dorsal to the cingulate sulcus that was posterior to the anterior commissure and anterior to the posterior commissure [31]. For more detailed information on the imaging pipeline please see S1 Text. We have made these Python scripts available for download (https://github.com/mcintyrelab).

### Force Matching Tasks

**Fig 1:**
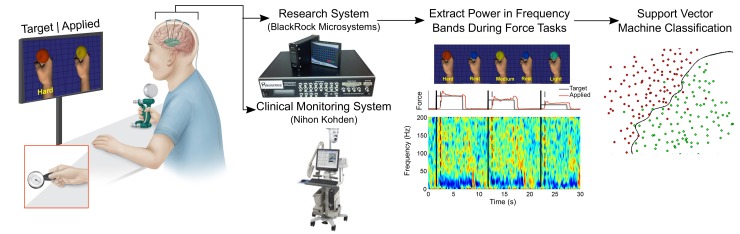
Experimental Setup. Participants had stereotactic depth electrodes placed for clinical epilepsy monitoring and were asked to perform force matching tasks in two different grasp configurations (power grasp and lateral pinch grasp–inset). Participants were prompted by a screen showing them the desired force level as well as their delivered level of force each represented as the color and size of a ball being squeezed on the screen. SEEG signals were split between the clinical monitoring system and the research neural recording system. Frequency band powers were calculated offline with a short-time-Fourier transform and used for discrete state classification.

Each participant performed a series of force matching tasks with the arm contralateral to electrode locations resting on a pillow or tabletop. An instrumented pinch gauge and power grasp dynamometer (Fabrication Enterprises Inc., White Plains, NY) were used to record exerted grasp forces (Fig 1). Hand orientation was consistent between both grasps to prevent any neural bias. Participants initially performed maximum voluntary contraction (MVC) tests in each of the two grasp configurations, and then force targets were set to 20, 30 and 40% of these MVC values. Force target levels were chosen to minimize fatigue but to also be high enough to elicit robust activation beyond the cortical baseline levels associated with simply holding the dynamometer. Participants were cued which force to exert by a split-screen visualization showing the target force level on one side and their current force level on the other. The visualization depicted a virtual hand squeezing a ball, with the grasp force level depicted by the changing ball size and color. Targets were presented for 5 seconds with 5 seconds of rest between successive trials. Each participant performed 5 trials of each of the 3 force levels during a single continuous block. Participants A, B, and D performed 4 blocks for power grasp trials and 4 blocks for pinch grasp trials (total of 20 trials of each force level for each grasp) while participant C performed 3 power grasp trials (15 trials for each force level) and 5 pinch grasp trials (25 for each force level).

Two participants (A and B) performed blocks where they only imagined making the desired force level without exerting any force on the sensor. These blocks were interspersed with the executed blocks to help the participants better imagine performing the desired forces. Visual cues were still given during these trials and the force sensor readings were monitored to make sure no forces were actually being applied.

### Recording, Data Preprocessing, and Feature Extraction

SEEG signals were split between the clinical Nihon Kohden (Irvine, CA) system and the research recording system using two 64-channel touch-proof connector splitter boxes (Blackrock Microsystems, Salt Lake City, UT). The experimental setup used a Neuroport data acquisition system (Blackrock Microsystems) to record signals at 2 kHz from up to 128 channels depending on the participant. Electrodes were hardware referenced to a depth electrode contact in a different anatomical region of the brain that was not related to seizure generation. SEEG signals were also common average referenced by grouping contacts passing through similar tissue structures and on the same electrode to reduce the large amounts of ambient noise from the hospital equipment. Particularly noisy channels were excluded from the common average.

The powers in six frequency features (δ/θ: 0-6Hz, α: 6–12 Hz, β: 12–30 Hz, γ: 30–50 Hz, and two high γ features: 70–110 and 130–170 Hz) were extracted from each channel after preprocessing using a short-time Fourier transform in 1 Hz frequency bins with a temporal window size of 512 ms and an update of 50 ms. For each 1 Hz frequency band, power was converted to a z-score for normalization. The local motor potential (LMP)/Low-frequency content(LFC) was also calculated as a feature for comparison with frequency power features. The LMP was computed in this study using a 2nd order Savitzky-Golay filter with a 250 ms window similar to Pistohl et al. (2012)[27]. The average z-score in each of the six frequency features as well as the LMP from each electrode channel made up the feature matrix (252 features for participants A and B, 224 features for participant C and 420 features for participant D). The delta and theta bands (δ/θ) were grouped together as performance for the two were similar when investigated separately and also to compare this feature to the LMP performance which had a similar frequency range but was in the time domain. The high gamma frequencies have been shown to not be individual frequency bands but actually a broadband change in power [32,33] but they were divided into different features for this study to avoid the 60 Hz noise band and its harmonics.

To determine statistically significant event related synchronization and desynchronization modulation peaks of each frequency feature, the maximum modulation/demodulation from baseline was found during a time window of -1 seconds to force onset. The maximum change was then compared against a baseline period of -3 to -1 seconds prior to force onset using a two-sample t-test with a very conservative p value of 0.002.

### State Classification

Cortical discrimination of grasp force states was assessed using a two-stage, cross-validated support vector machine (SVM) implemented with the libSVM package for Matlab [34]. A time window of -400ms to +400ms centered on force onset was used to average each frequency band feature for classification. For the rest trials, the data was centered on 1 second into the target trial to allow the channels to return to resting states after the force trials. SVM parameters ‘C’ (misclassification penalty parameter) and ‘δ’ (radial basis function kernel parameter) [35] were optimized inside of the inner cross-validation level before being tested on naïve data in an outer cross-validation level to see how well the model generalized. This process was used to investigate both single-feature and multiple feature classification accuracies. For the multiple feature trials, a sequential forward selection process was used to optimize which features to select along with the SVM parameters on the inner cross-validation level before being tested on the outer level. To mitigate the chance of model overfitting, the feature selection was halted when the increase in performance was not significantly better than the previous performance. Classification performance varied depending on how data was partitioned in the cross-validation so the two-stage cross-validation was repeated 10 separate times using different random partitions and the accuracies were averaged to give a more accurate representation of overall performance. The classification accuracies were then tested for significance above chance using a binomial test and adjusting *p*-values for multiple comparisons using the false discovery rate proposed by Benjamini and Hochberg [36]. The false discovery rate is less conservative than a Bonferroni correction and was set to 5% for this analysis.

## Results

### Localization of Electrode Contacts

**Fig 2:**
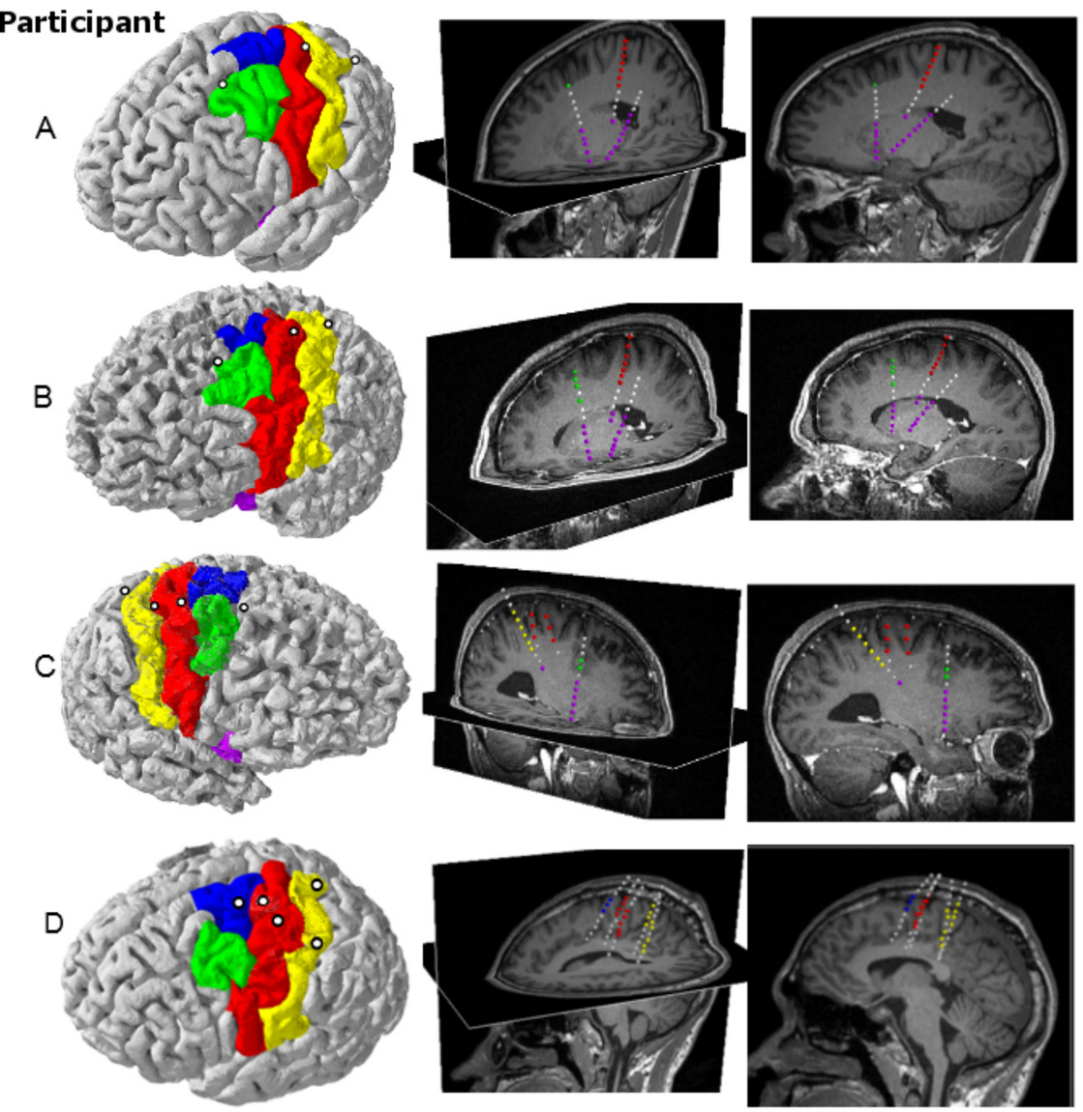
Locations of electrodes. Each row corresponds to an individual participant (A-D). *Left*: Participant specific cortical surface reconstructions (red: motor cortex, yellow: primary sensory cortex, green: premotor cortex, blue: supplementary motor cortex, purple: insular cortex) and electrode penetration sites (white circles). *Middle*: Oblique views of sagittal and axial MRI slices showing the electrode trajectories. Electrode contacts are color-coded according to the cortical region they pass by or through. The MRI slices shown are located as close to the electrodes as possible without obscuring any of the contacts. *Right*: Sagittal views showing the actual depths of the electrodes.

Fig 2 shows the locations of the electrode contacts passing through sensorimotor areas. All participants had at least one electrode passing through the arm/hand area of motor cortex. Participants A through C had motor electrodes placed close to central sulcus while participant D had their two electrodes placed much more centrally on the precentral gyrus. Each participant also had an electrode placed more anteriorly through the superior frontal sulcus except participant D who had his electrode placed through SMA. Finally, at least one electrode was placed by or through the primary sensory cortex although two participants (A and B) had their contacts too deep to be able to record from sensory cortex. Participants A, B and C had several contacts reaching the anterior and posterior insular cortex as well. To see the specific positions for these participants’ contacts, see S2 Fig.

### Modulation of Cortical Frequency Bands during Executed Grasp Force

**Fig 3:**
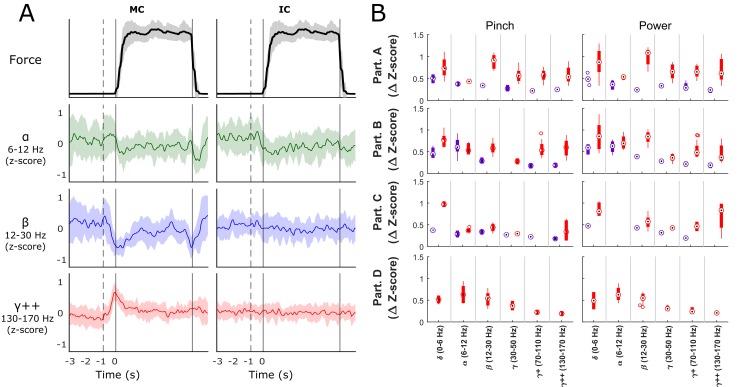
Signal Modulation during Force. Panel A shows average z-score modulation over time on one motor cortex (MC) channel and one insular cortex (IC) channel in the alpha, beta and highest gamma features during hard force tasks. The solid vertical line corresponds to force onset with the dotted vertical representing the average target cue time. Shaded regions indicate confidence intervals for the signal means of each frequency band. Panel B shows the mean ± standard deviation depth of modulation (change in z-score from baseline) for statistically significant MC (red bars) and IC (purple bars) channels, organized by participant (rows) and by frequency band (x-axis) for each of the two grasping configurations. All participants showed significant modulation in the both motor and insular cortices (excluding D who did not have electrodes placed in the insula).

Several SEEG channels showed obvious modulation during force trials (Fig 3). Fig 3A shows average modulation for an example electrode located inside of central sulcus of motor cortex, and a sample electrode residing in insular cortex (Participant B, hard power grasping trials). The motor channel shows the characteristic event-related desynchronization causing an increase in high gamma frequency power and decrease in beta band power at the onset of force execution, consistent with other field potential literature [26,37]. The increase in the high gamma frequency was largest in the period directly around force onset and quickly decreased afterwards (see S4 Fig for brain region specific task averaged spectrograms). Both alpha and beta activities of the motor channel exhibited an initial movement-related desynchronization at the onset of force execution, followed by either synchronization or steady-state activity during the force hold period, and finally a second movement-related desynchronization during force offset.

Fig 3B shows boxplots of channels with statistically significant ERD/ERS patterns during any of the force trials. The x-axis is grouped by frequency bands and the y-axis is the corresponding z-score magnitude of synchronization/desynchronization. Red boxes correspond to motor cortex contacts while purple correspond to insular cortex contacts. Rows are for each of the four participants and the two columns show results for pinch and power grasp configurations, respectively. All participants exhibited significant modulation in motor cortex contacts. All 3 participants with insular contacts (A-C) showed statistically significant delta/theta and alpha modulation (t-test, p < 0.002) during force tasks but only participant B had magnitudes of modulation comparable to that observed in motor cortical electrodes.

### Significantly Modulated Cortical Locations and Frequency Bands

We examined the amount of information the modulation of each frequency band in insular and motor electrodes conveyed for discrimination of force grasp states, through a support vector machine. For each grasp force trial, activity in each band was represented by the average power in a -400 ms to 400 ms window centered around force onset. This time window was chosen as it contained areas of greatest modulation in band powers as seen in Fig 3A. Average activity during each rest period was determined over a 600 ms to 1400 ms time window post force-offset to ensure that the neural signals had returned to baseline levels. We ensured that the same number of rest trials were included as each other force class, so as not to bias the prior probabilities of the classifier, and potentially bias more classified states to one class over another. SVM models were investigated for discrimination of the resting state versus all grasp force states as one class (2 state classification), as well as resting state versus the light and hard grasp force states (3 state classification).

**Fig 4:**
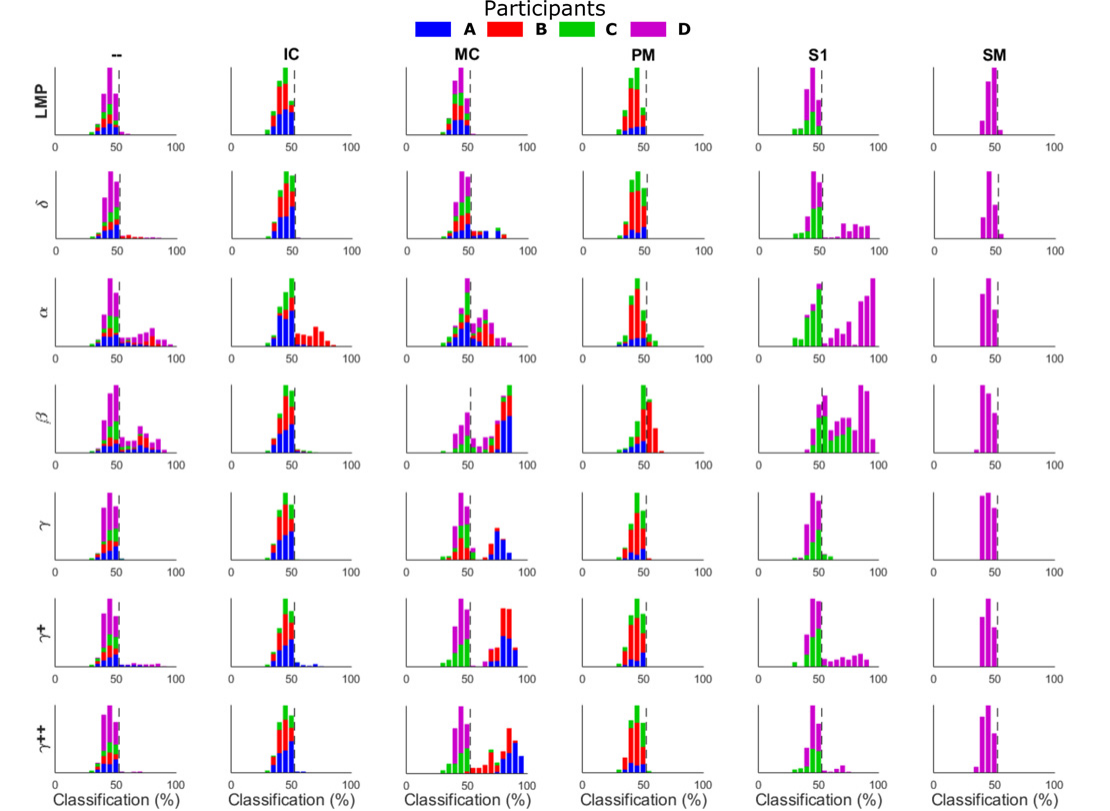
Classification density for single features. Histograms show the distribution of feature classification accuracies for force versus rest classification. These bar values represent the single feature classification accuracies across 10 repeats of the cross-validation and are organized by brain region (— = White matter tracts, IC = Insular Cortex, MC = Motor Cortex, PM = Premotor Cortex, S1 = Primary Sensory Cortex, and SM = Supplementary Motor Area) and feature (LMP = Local Motor Potential, δ/θ = 0–6 Hz, α = 6–12 Hz, β = 12-30Hz, γ = 30–50 Hz, γ+ = 70–110 Hz, γ++ = 130–170 Hz). Colors correspond to each participant (blue = A, red = B, green = C, and purple = D) with pinch and power grasp configurations combined. Dotted line (52.67%) shows the high end of the 95% confidence interval for chance classification. All participants had motor cortical features that could be used for discrimination of force versus rest in contrast to other brain regions which were more participant specific.

Fig 4 shows histograms of the distribution of single feature classification accuracies during rest versus force (2-state) discrimination. The histograms are arranged by feature frequency band (rows) and by brain region (columns) with different colors representing each participant. The dotted line shows the upper limit of a 95% confidence interval of chance classification after a permutation test, so bars to the right of this line represent features with movement related modulation for discriminating rest vs force statistically beyond chance (t-test, p < 0.05). All four participants had statistically significant discrimination of rest vs. force based upon activity in the motor cortex (MC) although the frequency bands with the most information varied between participants. All four participants exhibited beta modulation that allowed for statistically significant discrimination of rest vs force states. Participants A, B and C all had electrodes which exhibited movement-related high gamma modulation that allowed for statistically significant rest vs. move discrimination. Of the three participants with insular cortex contacts, only B had any features (alpha band) which exhibited grasp related modulation for rest vs force classification. Electrodes located in sensory cortex (participants C and D) exhibited movement related modulation in the alpha, beta, gamma, and high gamma frequency bands for above chance discrimination of rest vs force. Premotor cortex (PM) beta band contained discriminatory information for participant B but not A or C, while the supplementary motor area (SM) did not exhibit force related modulation (participant D only). Several contacts that appear to be placed through white matter tracts (—) between motor cortex and insular cortex and sensory cortex and insular cortex showed significant levels of modulation in the alpha and beta frequencies for all participants. Finally, the time domain local motor potential (LMP) features did not yield any significant classification results, contrary to other studies. Classification results further organized by particular electrode contact can be seen in S1 Fig.

### Classification of Executed Force States

**Fig 5:**
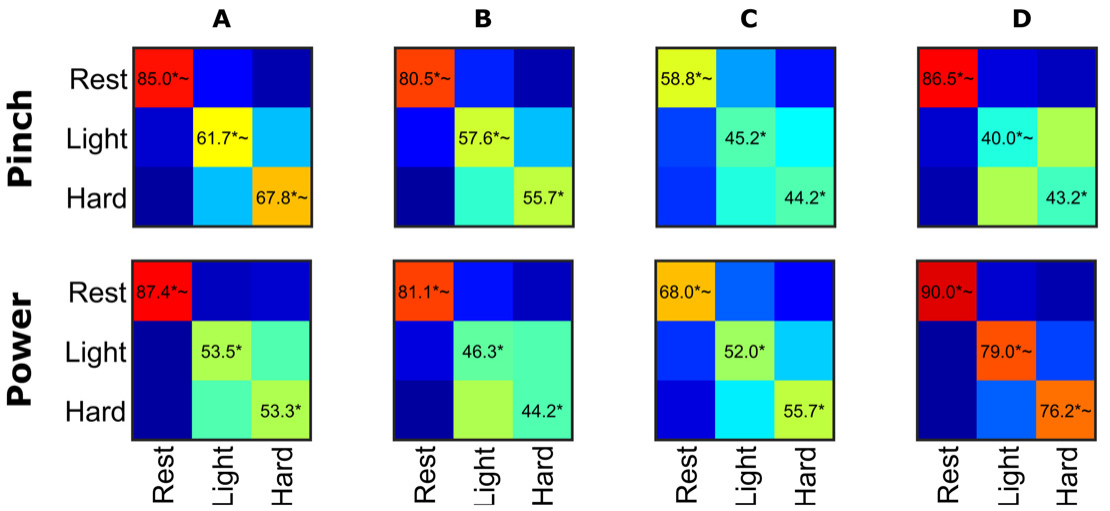
Rest versus Light versus Hard Classification Accuracies. Confusion matrices of light versus hard versus rest classification for each participant (columns) in the lateral pinch and power configurations (rows) using sequential feature selection and two levels of cross-validation. Features were averaged across a time window of -400 ms to +400 ms centered on force onset or 1 second after the rest cue. All participants showed the ability to differentially modulate neural activity between rest and force with high accuracy, but were less adept at differentially modulating neural activity between force levels. * denote significance above chance classification (1 in 3) using false-discovery rate adjusted p-values < 0.05 while ~ denotes a more stringent significance from 50% to see if light vs hard forces were significantly different.

We further investigated whether multiple grasp force levels could be discriminated beyond simply rest vs force. SVM classification of cortical modulation using sequential feature selection was not able to discriminate beyond chance between 10%, 20%, and 40% MVC for any participant or grasp configurations. Upon closer inspection of each participant’s ability to correctly modulate their force level to the desired target, it was observed that when participants were instructed to execute 30% MVC grasp force the recorded force values often overlapped the 20% or 40% MVC levels. Thus we attempted state classification of just the light and hard force levels (20 and 40% MVC which had more separation) plus rest. Fig 5 shows average confusion matrices for rest versus light versus hard forces. The confusion matrices were averaged across 10 trials of cross-validation to give a better approximation of performance. The SVM was able to produce overall statistically significant discrimination (adjusted for multiple comparisons using FDR) of light versus hard versus rest trials for all participants but for many participants this was due to the high accuracy to which the rest trials could be separated. To further investigate the level of discrimination, the classification rates were compared to two different chance levels: 1-in-3 (33.33% significance indicated with ‘*’ in Fig 5) and 1- in-2 (50%, significance indicated with ‘~’ in Fig 5). The second more stringent chance level percentage was chosen to show if light versus hard were discriminable from each other enough to reach significance above chance without the added benefit of having a third, well-discriminable class lowering the chance level. Based off of this criteria, the classifier was able to discriminate light and hard force levels for some participants in certain grasps such as power grasp for participant D and pinch for participant A.

### Classification of Imagined Force States

**Fig 6:**
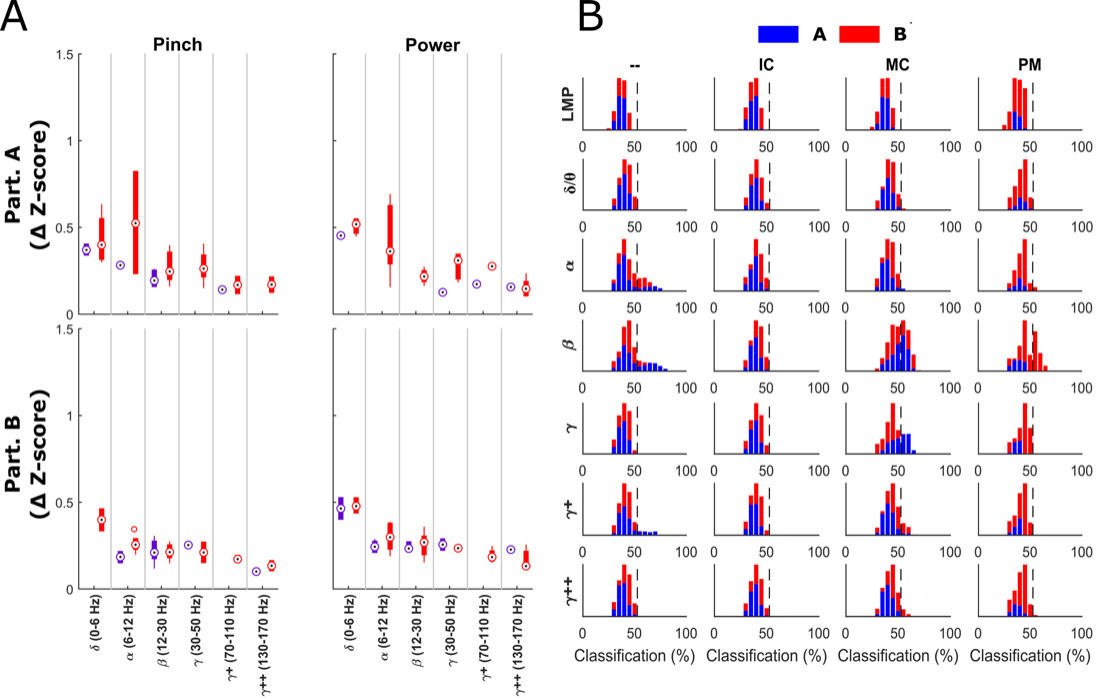
Imagined single feature modulation. Panel A shows the mean ± standard deviation depth of modulation (change in z-score from baseline) for statistically significant MC (red bars) and IC (purple bars) channels, organized by participant (rows) and by frequency band (x-axis) for each of the two imagined force grasping configurations. Modulation was lower as a whole during imagination compared to Fig 3B with the exception of the alpha band for participant A. Panel B shows single feature classification distributions for imagined force versus rest trials. Participant A is shown in blue and participant B is shown in red. The chance level is shown as the dotted line. Histograms are arranged by frequency band (rows) and by brain region (columns).

We also examined to what extent insular and motor cortical bands modulated their activities during tasks of purely imagined grasp force production in two participants (A and B). Fig 6A shows the boxplots of significant insular and motor cortical modulation for each participant during the first 2 seconds after target presentation. Compared to modulation during actual force grasp production (Fig 3B), modulation during purely imagined movements is decreased in magnitude in the insular and motor cortices, with the exception of the alpha band in motor cortex for participant A. The alpha band showed higher modulation values from baseline than during executed grasp force production. Of note is that imagined force related modulation is evident in insular cortical signals, predominantly in the lower frequency bands (< 30 Hz).

Fig 6B shows the single feature performance for classification of imagined “force” versus rest with both participants exhibiting significant beta modulation in the motor cortex that could be used to discriminate imagined force versus rest. The premotor contacts for participant B also exhibited statistically significant classification in the beta band just as in the executed forces. Participant A also exhibited modulation of the gamma band of motor cortex. No force classification information was able to be extracted from the insular cortex for either participant, however.

**Fig 7:**
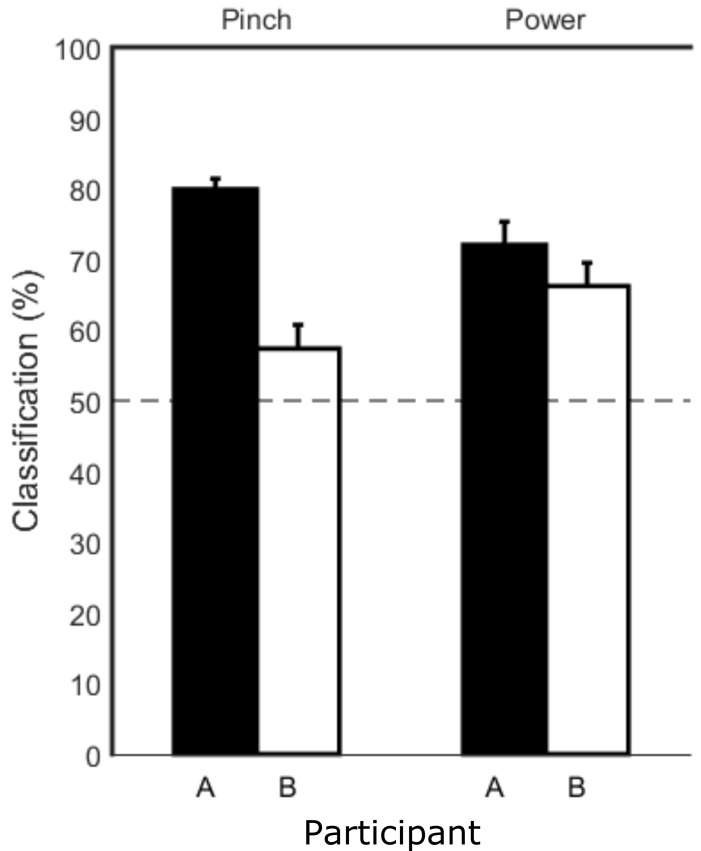
Average classification of imagined force versus rest. Bars show the average classification performance across 10 sets of cross-validation. The dotted horizontal line shows prior probability level (50%). The SVM was able to achieve significant classification of imagined force versus rest for both participants (A = black, B = white bars) and in both grasp configurations (pinch and power).

The modulation during imagined force trials was significant enough to give above-chance classification of imagined move versus rest using sequential feature selection as can be seen in Fig 7. Classification of light versus hard imagined forces versus rest did not yield above chance results. Since there was no force onset during the imagined trials and other studies have suggested that motor imagery tasks follow similar time courses to their executed counterparts [38], the average force onset time from the executed trials was used to center the frequency band windows used in the SVM.

## Discussion

The goal of this work was to investigate whether SEEG electrodes could record neural activity from sulci and deep cortical structures related to hand grasp force. The results show that SEEG electrodes are able to record signals from the insular cortex and inside of central sulcus that correspond to motor activity. These structures are usually inaccessible to other invasive brain recording electrode technologies such as ECoG grids or standard 1–1.5mm penetrating microelectrodes. This is the first study to our knowledge to investigate whether SEEG signals taken from the sulci and insular cortex could be used to discriminate executed and imagined grasp force levels.

### Spatial Locations and Frequency Bands of Interest

Several cortical locations showed modulation when comparing force generation trials to resting trials. These structures included cortical tissue of the motor wall of central sulcus from the fundus all the way up to the top of the gyri, as well as, different portions of insular, premotor and sensory cortices. While there still is some debate about the relationship between electrophysiological and BOLD recordings from inside of sulci due to the presence of blood vessels, our results do correspond with several fMRI imaging studies which show that much of the BOLD signal increases for hand and finger movements occur inside of the central sulcus [1,2]. Other studies showed BOLD activity in insular cortex during grasping [4,5]. We were not able to establish any modulation useful for multi-state classification of force level from insular cortex, but participant B did show obvious signs of alpha modulation in both the anterior and posterior insular cortices that were useful for classification between force production and resting.

The modulation observed in the insular cortex of participant B was found predominantly in the alpha band for both pinch and power grasp configurations. This is in contrast to the motor contacts which showed modulation in almost all of the bands (see S4 Fig for more comparison between brain regions and frequency modulation). ERD of the alpha band was suggested to be related to release from inhibition by Klimesch (2012)[21]. The modulation in motor cortex also had a much higher z-score associated with it implying that the level of activity was higher and/or more widespread. This would be consistent with imaging studies that usually show much higher activity in the motor cortex than in the insular cortex during force tasks [6–8,39]. The lower imaging activity in insular cortex may be because there are fewer neurons there active during the force tasks which would be consistent with the smaller amounts of modulation seen on insular contacts. This smaller network of neurons in the insular cortex could be involved with releasing certain portions of the motor network from inhibition and allowing movement as suggested by the ERD of the alpha band.

The modulation in insular cortex was found on electrode contacts in both the posterior and anterior insular cortex. Upon closer inspection of the region of insular cortex shown to be related to hand and finger movements by Mutschler et al. (2009)[5], the region was located on the posterior portion of the anterior insula, just in front of the central sulcus close to the center of the insula. The electrodes placed clinically to target the middle of the insular cortex in our study were just slightly too superior to reach this same region as can been seen in S1 Fig. This could partially account for the fact that modulation was not observed in the other participants. Another possible reason was that the above mentioned insular studies found in Mutschler’s meta-analysis had participants either opening and closing hands or moving their fingers and did not have them directly modulate force levels.

Some channels that appear to be in white matter tracts recorded movement-dependent modulation. There is some level of inaccuracy when coregistering pre-op MRI and post-OP CT scans as well as automated grey matter parcellations so it is possible that these electrodes might actually be close enough to be recording from surrounding grey matter. Without fully knowing the recording distance of these SEEG contacts (modeling studies of similarly sized deep-brain stimulating electrodes suggest this distance could be up to a few mm [40,41]) it is hard to tell for sure whether they are recording from surrounding tissue or actually able to pick up white matter signaling. See S1 Fig for an example of an electrode contact in white matter that was able to record alpha and beta modulation in participant B.

The frequency features found to deterministically modulate during force versus rest trials were the high gamma bands (70-110Hz, 130-170Hz) and the beta band (12-30Hz). Participants B and D also exhibited modulated activity in the alpha band. When looking at force discrimination tasks and excluding sensory cortex from participant D, however, only high gamma modulation resulted in classification accuracy greater than chance. This is consistent with other published literature showing that high gamma bands carry movement specific information while beta band modulation is predominantly useful for discrimination of rest versus movement activity [42–46]. The largest event related potentials were observed during the force transition periods.

The peak in modulation can easily be seen in participant B’s spectral traces in Fig 3A at the beginning of each force trial. These results correspond with fMRI results that show an increase in cortical activity during dynamic force trials rather than isometric force trials [9,39]. More force transition periods during dynamic trials could increase the activity of the corresponding neuron populations. Using EEG, Jochumsen et al. (2013)[47] showed that the magnitude of change in the movement-related cortical potentials (MRCP) increases more with fast force changes than slower force changes even at two different force levels. The MRCP values also had their peak magnitudes at movement onset and quickly decreased when the force was held constant, which is similar to this study. In some cases a second increase in high gamma power was observed at the end of the force generation trials which might correspond with an active opening rather than a passive relaxation of the hand.

Both participants still showed significant levels of modulation in both motor and insular cortices during imagined force execution. During the imagined force trials, the magnitudes of modulation were much lower than during the executed trials. This decrease in modulation amplitude is consistent with surface ECoG results seen in Miller et al. (2010)[48].

Interestingly, the LMP was not able to achieve statistical significance of force versus rest classification. This is in contrast to several recent ECoG papers that showed LMP to be their most useful feature for classification of grasp forces [26,27]. Perhaps the LMP signals are only a cortical surface phenomenon? More investigation would need to be done to make a definitive decision.

### Force Classification

Using only a single feature, several frequency bands across multiple contacts could be used to achieve classification accuracies well above chance for rest versus force classifications. It was not possible to achieve statistically significant classification of 3 force levels even using multiple features, however. Also, even though Figs 3B and 6A show frequency bands in insular cortex with significant modulation, this did not always coincide with the ability to use these features for classification as in Figs 4 and 6B. This difference was due to the fact that the maximum modulation values did not always occur within the -400 to +400ms window around force onset and that the features used in the classification were averages of the values during this time. The average values taken during this time window were apparently not significantly different than the values taken during the resting periods. Possible experimental confounds that led to lower classification rates include participant difficulty in executing the instructed grasp force targets, significant environmental noise on smaller contacts (participant C), and non-optimal electrode contact sizes.

The force targets chosen may have been too close to be differentiable using the field potentials and a larger gap in percentage of MVC might be necessary to achieve higher success in discrimination of force state. The force levels generated by the participants often overlapped with other force target levels especially when a participant would overshoot and undershoot before settling close to the target force level. There was also a lot of variability in the signals even during the same force tasks. This is most likely due to the fact that the magnitude of the signal modulations can vary considerably depending on the level of focus or attention given to the task [49] as well as the speed at which force is applied [47]. Participant C had much more environmental noise present during his experiments than the other participants and this would explain why the only features that could give greater than chance classification of move versus rest were in the lower frequency bands which usually have a higher signal to noise ratio.

A potential limitation of the current macro-contact depth electrodes is that the contact size may simply be too large (sampling too large of a neural population) to be able to distinguish between higher resolutions of motor control associated with smaller cortical networks. Kent and Grill (2014) [50] modeled the recording region of DBS electrodes with respect to contact size and found that longer contacts and/or electrodes with wider diameters both showed decreased signal amplitudes due to spatial averaging. Decreases in the recorded signal amplitudes reduce the signal to environmental noise ratio, thereby reducing the ability to extract movement-related information from signals for state classification. Lempka and McIntyre (2013)[40] and Lindén et al. (2011)[41] also modeled electrodes and cell clusters and found that the reach of local field potential (LFP) signals can extend several millimeters but is dependent on the synchrony and morphology of the cell groups. A large recording radius, on the order of millimeters, prolongs long-term signal stability in the presence of electrode encapsulation and/or neuronal death, but it also means that a large collection of neurons are being sampled. The larger the group of neurons, the greater the likelihood of asynchronous modulation between larger cortical networks, resulting in destructive interference and a loss of ability to distinguish higher resolution states of motor control. Smaller electrode contacts, including microwire electrodes with an appropriate amplification headstage, may be more advantageous for differentiating finer adjustments of force.

Even though the electrode locations varied between participants in this study due to clinical necessity, there was a large overlap in cortical regions that were sampled. All four participants had contacts placed in motor cortex, although participants A, B and C had electrodes passing through central sulcus and D had two electrodes passing through the gyrus, one in arm and hand area and the other in leg area. The fact that participant D’s motor contacts passed through the gyrus rather than by the central sulcus might account for the reduced classification ability from these electrode contacts. Participants A, B and C all had electrode contacts passing through the posterior and anterior insular cortex as well. Participants C and D also had electrode contacts that passed through sensory cortical tissue. Looking at the power spectral density plots in S3 Fig show that the frequency power cutoff varied between brain regions with motor cortex having the widest band and white matter having the shortest. These power cutoffs show the noise floor of the given contact [33] and could be related to different impedances at the contacts. The lower cutoffs might explain why some of the brain regions only recorded significant modulation in lower frequency bands since these higher band modulations could have been lost in the noise.

The modulation shown on all participants in motor cortex and sensory cortex for participants C and D is consistent with numerous other studies. The recording of force-onset related signals from the insular cortex in participant B, however, have not been reported in the literature to our knowledge. Imaging studies have implied its involvement in hand movement and force tasks but since it is hard to reach with other intracranial electrodes, recordings from it have not been demonstrated previously. The desynchronization of activity in this region in the alpha frequency band could mean that the insular cortex is involved with releasing other networks from inhibition. The activity in different regions of the insular cortex could be related to different networks as well. It has been proposed that the posterior insular cortex is largely related to viscerosensory and somatosensory information [51] while the anterior insular cortex has shown activity in numerous other tasks such as task-level control and focal attention [52], emotion, error-processing, [53] and eye and hand movements [5,15]. Further electrophysiological investigation of the insular cortex from smaller subregions using depth electrodes with smaller contacts could help elucidate more of the insula’s role in processing of information.

### Benefits of SEEG Depth Electrodes

Depth electrodes have the potential to be a new technology for investigation of neural activity from deeper brain structures in awake human participants. These electrodes allow for recording from deeper cortical structures such as the insular cortex and inside of the sulci which are difficult to approach using other invasive recording technologies. Our results corroborate with imaging studies that show force related modulation within these deeper structures, and specifically insular cortex. The current gold standard technology of silicon penetrating microelectrodes achieve fairly high levels of performance by recording individual neuron signals but only penetrate 1–1.5mm into the surface of the brain which would not be able to reach these deeper structures. While there are some reports of ECoG contacts implanted inside sulci [14,54,55], it is still difficult to place them into deeper structures such as the insular cortex without major disruption to brain tissue.

The penetrating microelectrodes also have been shown to be prone to signal degradation over time [56–59]. SEEG electrodes are similar in size to DBS electrodes which have been shown to be able to perform chronic recordings up to 7 years [60,61]. However, these studies were only looking at low frequency signals (beta band, which are usually much larger in magnitude than gamma and high gamma signals. The fact that the electrodes were also being continuously stimulated could help with issues from encapsulation caused by adhered proteins and cells [62]. Due to both of these factors, the long-term potential for recording of high frequency signals from SEEG electrodes is unclear but there is evidence suggesting that it might be possible.

Subdural ECoG grids and penetrating microelectrode arrays both currently involve surgeries requiring craniotomies. SEEG placement is minimally invasive and requires only a minor stab incision and a small burr hole to access the intracranial space. Several studies have shown that SEEG implantation is at least as safe craniotomy for implantation of electrode arrays [63–69]. Compared with craniotomy, SEEG implantation is associated with much faster postoperative recovery, less time required for tissue healing, and smaller scars, all of which could affect acceptance of this technology.

### Implications for the Field

Further testing is necessary to determine whether more discriminable force signals can be attained using depth SEEG electrodes with smaller contacts with more specific targeting of cortical structures. Smaller contacts may provide better accuracies than shown here and in the ECoG studies. New combination macrocontact/microwire depth electrodes could potentially provide the best of both worlds: high information throughput from single-unit recordings but also the more stable recordings of population dynamics from the field potentials. Placements of electrodes in this study were limited to anatomic structures that required clinical monitoring. By using fMRI to pinpoint exact cortical regions that modulate with desired tasks prior to surgery, performance might be increased. Deep brain stimulation electrodes appear to be tolerated by neural tissue well enough for years of implantation and depth electrodes are similar in size and material. It will be necessary to show the long-term viability of depth electrodes for chronic implants for long-term neurophysiology studies or brain-machine interfaces (BMI) but other field potentials have been shown to be stable for long periods of time. This study also corroborates with imaging studies that show force-related activity occurs inside the central sulcus and insular cortex. SEEG electrodes can be used to record activity from these brain regions which are difficult to reach with any other intracranial electrode technology. While not able to provide significant discrimination of different force levels with the electrode contact setup used in this study, SEEG electrodes were able to show significant modulation in the central sulcus and insular cortex between force and movement. The possibility of SEEG electrodes being able to record from white matter tracts should also be investigated further. These regions (intrasulcal primary motor cortex, insular cortex and potentially even white matter tracts) could prove useful targets for future BMI hand grasp systems if force level discrimination could be shown in future studies. The ability to record signals related to force activities when force is not actually being applied is also important for BMI in paralyzed patients. Though this study was not able to discriminate different levels of force during imagery, it was able to show discrimination of force imagery versus rest. This is important to the field because these signals were recorded from intrasulcal motor cortex during motor imagery which, to our knowledge, has not been shown before. It also lends support to the idea that force related activity can be found in motor cortex and that the changes in modulation are not just artifacts of sensory information changes. In conclusion, SEEG electrodes already being placed clinically for epilepsy monitoring also provide a unique platform for advanced electrophysiological study of deeper brain tissues in a wide assortment of tasks beyond just grasp force generation, such as decision making, and should be utilized to advance our understanding of these deeper structures.

## Supporting Information

S1 Text Supplemental Methods. (DOCX)

S2 Text Supplemental Results. (DOCX)

S1 Fig Spatial Locations of features. Electrodes are organized by clinical location they targeted (AI = Anterior Insula, MI = Middle Insula, PI = Posterior Insula, PS = Precentral sulcus side of primary motor, CS = Central sulcus side of primary motor, M_A/H_ = primary motor in arm/hand area, M_L_ = primary motor in leg area, S_A/H_ = sensory cortex in arm/hand area and S_L_ = sensory cortex in leg area). Square electrode contacts are color coded to match the cortical structures they pass by as shown in Fig 2 (insula = purple; motor = red, sensory = yellow; premotor = green; and SMA = blue. Uncolored contacts were not located in grey matter, either in white matter or outside of the brain) Colored circles represent classification accuracy of force versus rest for each electrode feature for the pinch and power grasp configurations. Small black circle represent features that were not statistically above chance (adjusted for multiple comparisons using false discovery rate. Circle color corresponds to classification accuracy. A motor contact is highlighted with a red square for each participant with the location of that contact shown on coregistered pre-op MRI and post-op MRI. Participant B also highlights the position of a contact in the white matter which recorded signal modulations in the lower frequency bands. It can be seen that all participants had some modulation during grasp force tasks in motor cortex, specifically from gray matter located within the sulci. Some participants also had recorded modulation in sensory cortex and white matter and one participant observed alpha band modulation in some insular cortex electrodes.
(TIF)

S2 Fig Insular Cortex Renderings along with electrode contacts. This figure shows the rendered insular cortex for Participants A-C. Electrode contact locations are shown as spheres color-coded to the brain regions they are passing through (red = motor cortex, green = premotor cortex, yellow = sensory cortex, purple = insular cortex). The insular cortex renderings are shown in yellow in order to make the locations of the purple insular contacts more apparent. The fMRI region of interest shown in Mutschler et al. 2009 [5] with the greatest activation likelihood estimates for insular cortex appears to be located in anterior insula close to the sulcus centralis insulae. Unfortunately this region was just out of reach of the insular electrodes placed for clinical monitoring.
(TIF)

S3 Fig PSD by region. Here is an example from Participant A showing the common average referenced power spectral density for single contacts in 3 different brain regions (MC = motor cortex, — = White matter, IC = Insular Cortex). The top row shows the raw PSDs while the bottom row shows the z-score normalized change in PSDs. Red traces show average resting values while blue shows the average force trial values -400ms to +400ms centered on force onset.
(TIF)

S4 Fig Average spectrograms from different regions. Here are example spectrograms from participant B with frequency on the y-axis and time on the x-axis showing average trial modulation for three different contacts located in different brain regions (MC = Motor cortex; IC = Insular cortex; WM = white matter). Spectrograms are the z-score normalized log powers across all force trials for each contact and the ranges are cutoff at a maximum of ± 0.5. It can be seen that the largest modulation occurs at the beginning of the force trial during force onset (dotted black line) for motor cortex but last longer and are contained in lower frequency bands for the insula and white matter contacts.
(TIF)
