# Supplementary material for: Contributions of Subsurface Cortical Modulations to Discrimination of Executed and Imagined Grasp Forces through Stereoelectroencephalography
Source: PLoS One. 2016 Mar 10;11(3):e0150359. doi: 10.1371/journal.pone.0150359 (PMC4786254; doi:10.1371/journal.pone.0150359)
Supplement: S1 Text — (DOCX) [file pone.0150359.s001.docx]

**S1 Text. Supplemental Methods**

All subjects underwent pre-operative scanning on a 3T MR system (MAGNETOM Verio, Siemens, Erlangen, Germany) at University Hospitals Case Medical Center. To localize the electrodes position, a post-operative CT scan was acquired (Brilliance iCT, Philips, Cleveland, Ohio or SOMATOM Sensation 16 or Cardiac 64, Siemens, Erlangen, Germany).

The DICOM images were de-identified using OsiriX (http://www.osirix-viewer.com) and then converted to the NIfTI (Neuroimaging Informatics Technology Initiative, http://nifti.nimh.nih.gov) file format using MRICron’s dcm2nii tool (http://www.mccauslandcenter.sc.edu/mricro/) to allow for image post-processing. Images were then visualized in FSLView to verify correct labeling and orientation. FSL’s fslreorient2sd tool was used to re-orient all images to the same orientation as FSL’s standard templates.

The post-operative CT was co-registered to the pre-operative T1-weighted MRI using FSL’s Linear Image Registration Tool (FLIRT). Parameters for the registration include a cost function of mutual information, rigid body transformation (6 degrees of freedom), and trilinear interpolation.

All processing and visualization was run on an 8-core, 64gb high-performance Linux-based workstation.
