# Supplementary material for: Contributions of Subsurface Cortical Modulations to Discrimination of Executed and Imagined Grasp Forces through Stereoelectroencephalography
Source: PLoS One. 2016 Mar 10;11(3):e0150359. doi: 10.1371/journal.pone.0150359 (PMC4786254; doi:10.1371/journal.pone.0150359)
Supplement: S2 Text — (DOCX) [file pone.0150359.s002.docx]

**S2 Text. Supplemental Results**

Fig 4 showed the histogram results of the single feature classification of move versus rest trials organized by feature and brain region for each participant. To further elaborate on the spatial locations of features, S1 Fig shows the average single feature classification results divided up by Grasp type as well as per individual contact. The figure also shows highlighted channels in co-registered pre-operative MRI and post-operative CT scans from 3 different planes. All 4 participants had electrode contacts in motor cortex containing features that could be used to give above chance classification of move versus rest. The figure also shows the location of one channel located in white matter that recorded modulating signals for participant A.

The power spectral density ranges varied by electrode region as can be seen in S3 Fig. The frequency range appears to go higher in motor cortex with insula slightly lower and white matter even lower than that before leveling off. In Miller et al. 2009 [33] it was shown that in ECoG signals this leveling off was due to reaching the noise floor for that particular electrode. This could affect the frequency band features if they are averaged into this noise floor but the PSDs do not appear to show much information in the higher frequencies anyway for regions besides motor cortex. The figure also shows that the modulation in the high frequencies is not separate bands but actually a broadband increase in power which was also described in other papers[32,33]. The figure also shows that when the frequency power is normalized (in this case using a z-score) then some of the spectral differences become more apparent, especially in the lower frequencies.

To show the power spectral changes in different brain regions the trial-averaged spectrograms for participant B were also plotted in motor cortex (MC), insular cortex (IC), and white matter (WM) (S4 Fig). The spectrograms are averaged across all force trials with 1 second of data prior to force onset (black line) to 3 seconds post force onset. You can see decreases in blue with increases in yellow with the majority of the modulation happening around force onset and slowly tapering off after that for motor cortex. In the insular and white matter tracts the modulation is much longer in duration and found in the lower frequency bands.

The power spectral density ranges varied by electrode region as can be seen in S3 Fig. The frequency range appears to go higher in motor cortex with insula slightly lower and white matter even lower than that before leveling off. In Miller et al. 2009 [33] it was shown that in ECoG signals this leveling off was due to reaching the noise floor for that particular electrode. This could affect the frequency band features if they are averaged into this noise floor but the PSDs do not appear to show much information in the higher frequencies anyway for regions besides motor cortex. The figure also shows that the modulation in the high frequencies is not separate bands but actually a broadband increase in power which was also described in other papers[32,33]. The figure also shows that when the frequency power is normalized (in this case using a z-score) then some of the spectral differences become more apparent, especially in the lower frequencies.
