# Supplementary figures and images for: Contributions of Subsurface Cortical Modulations to Discrimination of Executed and Imagined Grasp Forces through Stereoelectroencephalography
Source: PLoS One. 2016 Mar 10;11(3):e0150359. doi: 10.1371/journal.pone.0150359 (PMC4786254; doi:10.1371/journal.pone.0150359)

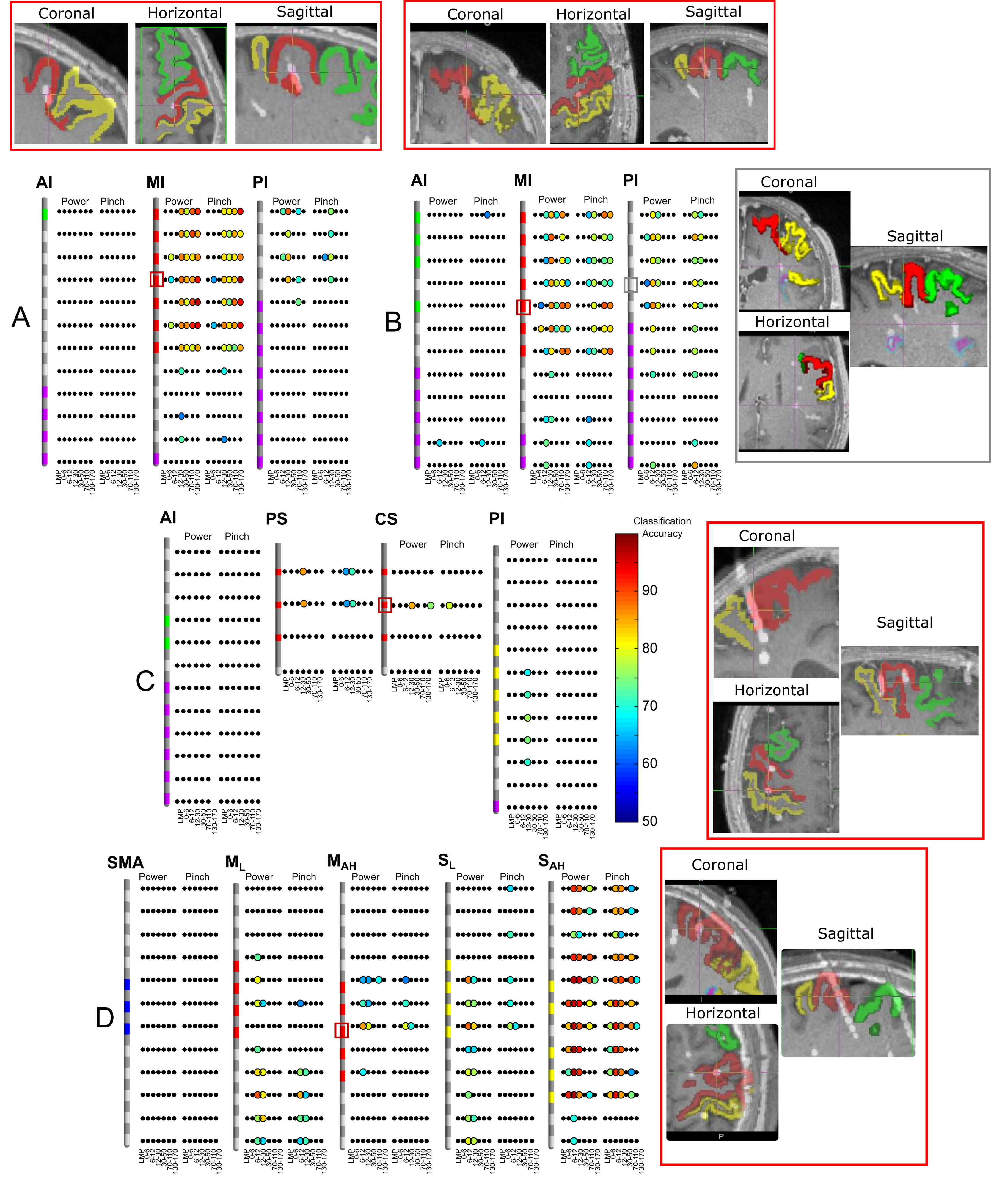

Supplement: S1 Fig — Electrodes are organized by clinical location they targeted (AI = Anterior Insula, MI = Middle Insula, PI = Posterior Insula, PS = Precentral sulcus side of primary motor, CS = Central sulcus side of primary motor, MA/H = primary motor in arm/hand area, ML = primary motor in leg area, SA/H = sensory cortex in arm/hand area and SL = sensory cortex in leg area). Square electrode contacts are color coded to match the cortical structures they pass by as shown in Fig 2 (insula = purple; motor = red, sensory = yellow; premotor = green; and SMA = blue. Uncolored contacts were not located in grey matter, either in white matter or outside of the brain) Colored circles represent classification accuracy of force versus rest for each electrode feature for the pinch and power grasp configurations. Small black circle represent features that were not statistically above chance (adjusted for multiple comparisons using false discovery rate. Circle color corresponds to classification accuracy. A motor contact is highlighted with a red square for each participant with the location of that contact shown on coregistered pre-op MRI and post-op MRI. Participant B also highlights the position of a contact in the white matter which recorded signal modulations in the lower frequency bands. It can be seen that all participants had some modulation during grasp force tasks in motor cortex, specifically from gray matter located within the sulci. Some participants also had recorded modulation in sensory cortex and white matter and one participant observed alpha band modulation in some insular cortex electrodes. (TIF) [file pone.0150359.s003.tif]

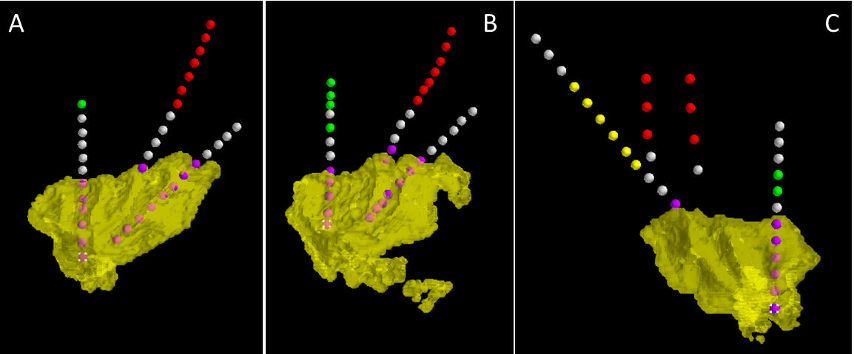

Supplement: S2 Fig — This figure shows the rendered insular cortex for Participants A-C. Electrode contact locations are shown as spheres color-coded to the brain regions they are passing through (red = motor cortex, green = premotor cortex, yellow = sensory cortex, purple = insular cortex). The insular cortex renderings are shown in yellow in order to make the locations of the purple insular contacts more apparent. The fMRI region of interest shown in Mutschler et al. 2009 [5] with the greatest activation likelihood estimates for insular cortex appears to be located in anterior insula close to the sulcus centralis insulae. Unfortunately this region was just out of reach of the insular electrodes placed for clinical monitoring. (TIF) [file pone.0150359.s004.tif]

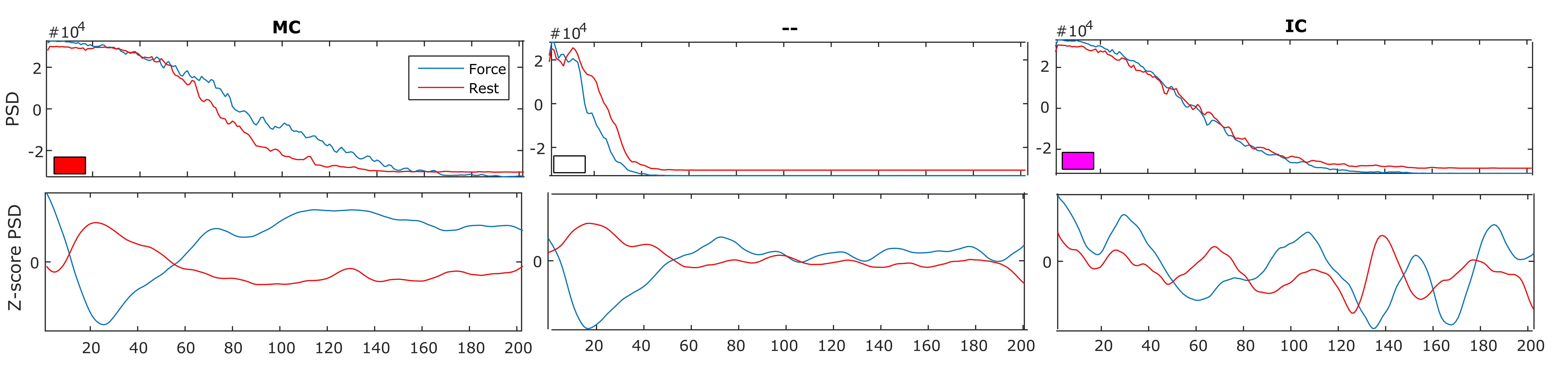

Supplement: S3 Fig — Here is an example from Participant A showing the common average referenced power spectral density for single contacts in 3 different brain regions (MC = motor cortex, — = White matter, IC = Insular Cortex). The top row shows the raw PSDs while the bottom row shows the z-score normalized change in PSDs. Red traces show average resting values while blue shows the average force trial values -400ms to +400ms centered on force onset. (TIF) [file pone.0150359.s005.tif]

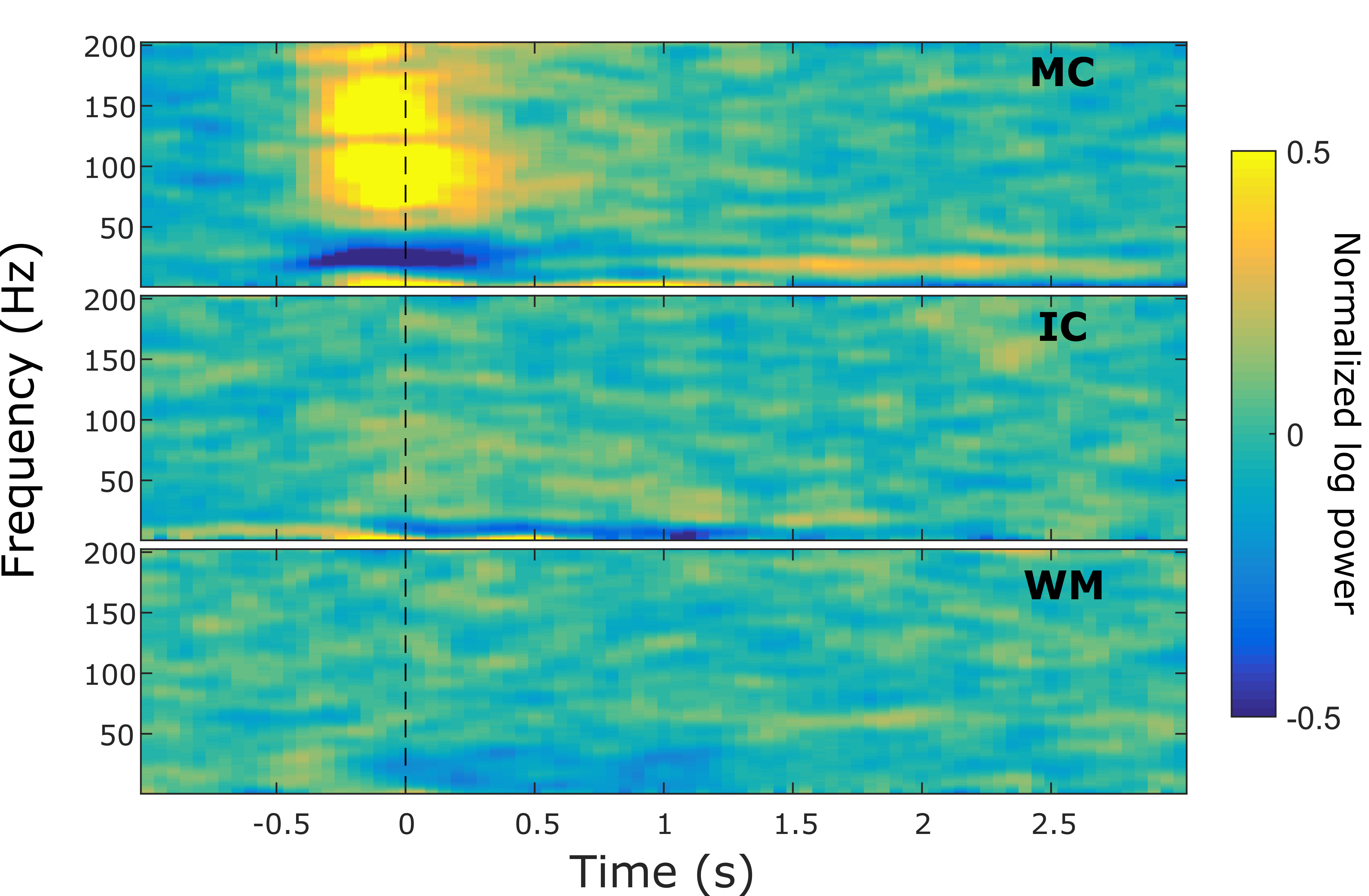

Supplement: S4 Fig — Here are example spectrograms from participant B with frequency on the y-axis and time on the x-axis showing average trial modulation for three different contacts located in different brain regions (MC = Motor cortex; IC = Insular cortex; WM = white matter). Spectrograms are the z-score normalized log powers across all force trials for each contact and the ranges are cutoff at a maximum of ± 0.5. It can be seen that the largest modulation occurs at the beginning of the force trial during force onset (dotted black line) for motor cortex but last longer and are contained in lower frequency bands for the insula and white matter contacts. (TIF) [file pone.0150359.s006.tif]
